# Feature Selection for the Interpretation of Antioxidant Mechanisms in Plant Phenolics

**DOI:** 10.3390/molecules28031454

**Published:** 2023-02-02

**Authors:** Taiki Fujimoto, Hiroaki Gotoh

**Affiliations:** Department of Chemistry and Life Science, Yokohama National University, Hodogaya-ku, Yokohama 240-8501, Japan

**Keywords:** chemical space, interpretation, structure–activity relationship, antioxidants, machine learning, feature selection

## Abstract

Antioxidants, represented by plant phenolics, protect living tissues by scavenging reactive oxygen species through diverse reaction mechanisms. Research on antioxidants is often individualized, for example, focusing on the evaluation of their activity against a single reactive oxygen species or examining the antioxidant properties of compounds with similar structures. In this study, multivariate analysis was used to comprehensively examine antioxidant properties. Eighteen features were selected to explain the results of the antioxidant capacity tests. These selected features were then evaluated by supervised learning, using the results of the antioxidant capacity assays. Dimension-reduction techniques were also used to represent the compound space with antioxidants as a two-dimensional distribution. A small amount of data obtained from several assays provided us with comprehensive information on the relationships between the structures and activities of antioxidants.

## 1. Introduction

Although oxygen is necessary for life, some oxygen in the body can become reactive oxygen species (ROS). Antioxidants are compounds that can delay, inhibit, or prevent the oxidation of materials by scavenging free radicals [1]. Oxidative stress occurs when the level of ROS becomes too high for the body’s antioxidant system to remain balanced. It plays a role in the development of chronic degenerative diseases, including coronary heart disease, cancer, and aging [2]. Therefore, antioxidants need to be included in a healthy diet. 

In this study, we considered antioxidants to be compounds that scavenge ROS by chemical reduction or physical energy transfer. The amount or ratio of ROS that they scavenge is known as the antioxidant capacity. This is typically determined as a value relative to different reference compounds in each assay. The reaction mechanisms and targets vary depending on the compound; therefore, many individual studies have examined the antioxidant capacities of compounds that have similar structures and mechanisms of scavenging ROS [3,4]. However, it is difficult to comprehensively analyze the structure–activity relationship (SAR) of compounds. To solve this problem, it is necessary to visualize the distribution of compounds according to their activity, as well as elucidate the relationship between the evaluation methods. The relationships among multiple antioxidant assays have been revealed by comparing the measured data in previous studies. For example, Mérillon et al. [5] compared the antioxidant properties of 30 food extracts using various assays. It is important to investigate the relationship between the molecular structure and antioxidant properties using multiple assays.

In recent years, the use of artificial intelligence has grown in terms of discussing quantitative SARs, particularly for small molecules [6]. This has been made possible through the development of many molecular descriptors and cheminformatics tool packages such as RDKit [7]. However, data analysis using a variety of descriptors as features can make it difficult to interpret the output. In particular, in chemistry, most experimental methods, except high-throughput screening, cannot provide large volumes of data. Insufficient data and an excessive number of features can often result in overfitting or the detection of false tendencies. Miyao et al. [8] predicted the enantioselectivity of organic reactions using a phosphoric acid catalyst with a small dataset containing extended connectivity fingerprints (ECFP) [9]. Their model showed a good predictive performance, thereby revealing the importance of two-dimensional structural information. ECFP typically represents a molecule by thousands of bits, which each represent the presence or absence of the corresponding substructure. However, the well-known ECFP is often folded to a fixed bit length, which causes bit collisions and a loss of interpretability [10]. It is, therefore, important to select an appropriate number of numeric features and perform a multivariate analysis to explain the desired characteristics.

Several feature selection methods have been previously developed, such as the filter, wrapper, and embedded methods [11]. Feature selection methods have been studied in the field of chemistry. For example, Wu et al. [12] applied the filter method to measure the 2,2-diphenyl-1-picrylhydrazyl (DPPH) radical-scavenging capacity. Their study used descriptors obtained from RDKit and decreased the number of features from 200 to 16 by conducting a *t*-test and discriminant analysis. As for the application of dimension reduction methods in chemistry, Bassoli et al. [13] showed the chemical space of bitter taste receptor agonists using *t*-distributed stochastic neighbor embedding, whilst only using fragments to describe the structural properties of the molecules. Furthermore, Süleyman et al. [14] developed ChemPlot, a Python library for chemical space visualization, using dimension-reduction techniques. This can visualize the distances between compounds in chemical spaces using 200 molecular descriptors instead of structural information. However, the activity cliff problem should be considered, in which structurally similar compounds have different potencies on the same target [15]. The two-dimensional representation of chemical properties using ChemPlot has become a useful way to avoid this activity cliff problem. This indicates that the structural information of molecules can sometimes be insufficient for describing chemical phenomena.

**Figure 1:**
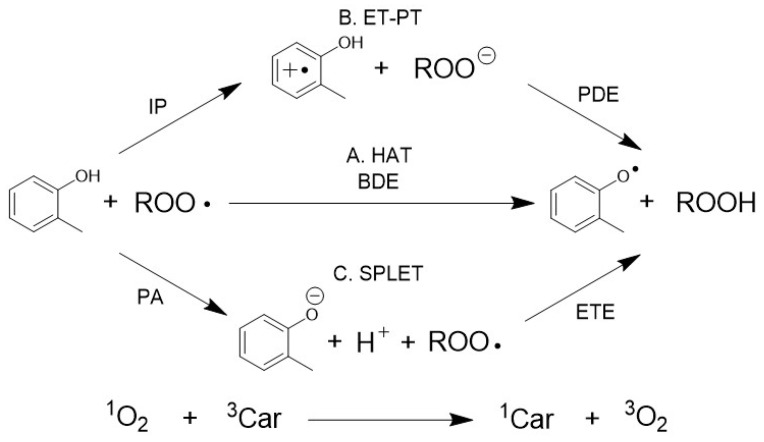
Well-known mechanisms of scavenging oxygen radicals and singlet oxygen. Pathway A shows the hydrogen atom transfer (HAT) mechanism, which is dependent on BDE. Pathway B shows the electron transfer–proton transfer (ET–PT) mechanism, which is dependent on IP and PDE. Pathway C shows the sequential proton loss electron transfer (SPLET) mechanism, which is dependent on PA and ETE. Singlet oxygen was quenched by physical quenching or charge transfer.

In this study, we conducted a multivariate analysis using the measured antioxidant data as a tool to describe SAR. We selected common features that could explain multiple antioxidant capacity indicators using partial coefficients and well-known reaction mechanisms. Common features were then prepared for each dataset that was used in the prediction task. We chose two methods to evaluate how common features described comprehensive information regarding antioxidants. One of these was the prediction of several targets using supervised learning, which is machine learning that has target values to predict, whilst the other method was the creation of compound distributions by dimension reduction. The quenching mechanisms of ROS have been identified in previous studies [16,17,18,19]. Several thermodynamic properties, including bond dissociation energies (BDE) and ionization potentials (IP), are useful indicators of the reactivity of a compound toward ROS. Therefore, the properties listed in Table 1 were obtained from quantum chemical calculations and added to the descriptors used in the multivariate analysis. Both molecular structures and thermodynamic aspects were used as explanatory variables to describe antioxidant properties. Peroxyl radicals are scavenged by the mechanisms depicted in Figure 1. Five thermodynamic properties are indicators of reaction mechanisms. These values were calculated using the formation enthalpies, H, using Equations (1)–(5).

(1) $$\mathrm{Bond}\;\mathrm{dissociation}\;\mathrm{enthalpy}\;(\mathrm{BDE})=H\;(\mathrm{Ar}\dot{O})+H\;(\dot{H})-H\;\left(\mathrm{ArOH}\right).$$

(2) $$\mathrm{Ionization}\;\mathrm{potential}\;(\mathrm{IP})=H\;({\mathrm{ArO}\dot{H}}^{+})+H\;(e^{-})-H\;(\mathrm{ArOH}).$$

(3) $$\mathrm{Proton}\;\mathrm{dissociation}\;\mathrm{enthalpy}\;(\mathrm{PDE})=H\;(\mathrm{Ar}\dot{O})+H\;\left(H^{+}\right)-H\;({\mathrm{ArO}\dot{H}}^{+}).$$

(4) $$\mathrm{Proton}\;\mathrm{affinity}\;(\mathrm{PA})=H\;\left({\mathrm{ArO}}^{-}\right)+H\;\left(H^{+}\right)-H\;\left(\mathrm{ArOH}\right).$$

(5) $$\mathrm{Electron}\text{-}\mathrm{transfer}\;\mathrm{enthalpy}\;(\mathrm{ETE})=H\;(\mathrm{Ar}\dot{O})+H\;(e^{-})-H\;({\mathrm{ArO}}^{-}).$$

We used five different assays for antioxidant capacity data: oxygen radical absorbance capacity (ORAC) [3], singlet oxygen absorption capacity (SOAC) [4], 3-(4,5-dimethylthiazole-2-yl)-2,5-diphenyltetrazolium bromide assay (MTT) [20], 2,2’-azinobis(3-ethylbenzothiazoline-6-sulfonic acid) radical cation decolorization assay (ABTS) [21], and DPPH radical-scavenging capacity [12]. Antioxidant capacity data for compounds from five different test methods were used for machine learning. Other in vitro and in vivo antioxidant assays are also known. For example, the cupric reducing antioxidant capacity method [22] and the ferric reducing ability of plasma assay [23] are known as in vitro methods. Additionally, the superoxide anion assay [24] and lipid peroxidation assay using 2-thiobarbituric acid reactive substances as markers [25] are known as in vivo methods. However, because the number of structure–activity relationship studies was small compared to those employing DPPH, ORAC, and SOAC, they were excluded from this study.

**Table 1:** Datasets used in this study. Five datasets were prepared for each task and were used for both supervised learning and dimension reduction. The remaining two without tasks were used only for dimension reduction. MEXT is a dataset consisting of food ingredients listed in the document published by the Japanese Ministry of Education, Culture, Sports, Science, and Technology.

Dataset	Task	Size	Target Chemicals
ORAC	Regression	70	Peroxyl radicals
SOAC	Regression	71	Singlet oxygen
MTT	Regression	71	MTT
ABTS	Regression	90	ABTS radical cation
DPPH	Classification	198	DPPH radical
Phytochemicals [26]		344	
MEXT [27]		109	

## 2. Results and Discussion

### 2.1. Feature Selection

Eighteen features were selected from the 113 candidate continuous value indicators obtained from the RDKit and PM7 calculations. Few dependencies were found between the selected features, and 18 features had no duplicates from either chemical or numerical perspectives. These features could be divided into four groups according to their chemical meanings, as shown in Table 2, and five thermodynamic properties obtained from PM7 calculations were included alongside five features of the polarity and electronic states. Six features were occupied by values representing bond distances and structural complexity. The remaining features represent the steric structures. The scatter plot matrices for each dataset are shown in Supplementary Figures S1–S7. The partial correlation matrices of the features in Table 2 for each dataset are shown in Supplementary Figures S8–S12. The partial correlation matrix was obtained from the inverse of the variance–covariance matrix. Each component represents the correlation between residuals obtained by subtracting the contributions of other features from the corresponding features. In this way, we confirmed that no dependency existed between the features.

### 2.2. Supervised Learning Using XGBoost

**Figure 2:**
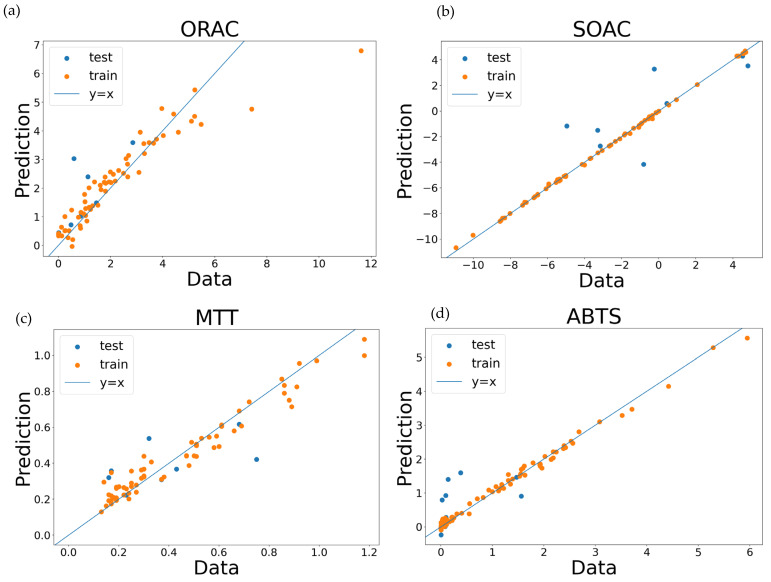
Prediction results at five regression tasks. The orange markers show training data, whilst the blue ones show the prediction of test data. The blue lines indicate where the plots are located when the predictions were correct. (**a**) The prediction performance when the ORAC dataset was used. (**b**) The prediction of SOAC values. (**c**) The prediction of MTT values. (**d**) The prediction of TEAC values included in the ABTS dataset.

**Figure 3:**
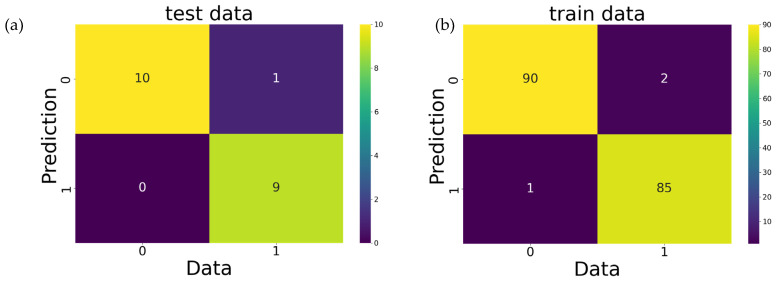
Confusion matrices for DPPH classification. (**a**) The classification performance of test data. (**b**) Plot indicating how well the classification model was trained. A score of 0 means no antioxidant capacity according to the DPPH assay, whereas 1 means that compounds in the area were effective.

The machine learning models used here were screened from nine algorithms using EvalML [28], an automated machine learning library. Because extreme gradient boosting (XGBoost) [29] showed stable predictive performance, it was used as an algorithm for supervised learning models. XGBoost is a tree structure-based algorithm wherein branches are generated by the relationship between feature values and thresholds, outputting predictions at the ends. During training, the model is improved by sequentially adding tree structures, such that the difference between predictions and true values approaches a minimum. The evaluation metrics for the prediction performance of XGBoost models are listed in Table 3. Smaller values for mean absolute error (MAE), root-mean-square error (RMSE), and MAE divided by the standard deviation (MAE/STD) used in the regression task evaluation indicated a stronger prediction performance. For the classification task evaluation, a lower binary cross-entropy and higher accuracy also indicated a better prediction. Plots consisting of the data and predicted values are shown in Figure 2a–d, whilst the confusion matrix of both the training and the test data for the classification task is shown in Figure 3a,b. It was confirmed that the predictions obtained from XGBoost in this study did not deviate from the actual values observed in the data. 

The feature importance was obtained from the XGBoost models for each task and is shown in feature_importance.xlsx within the Supplementary Materials; features with higher importance for each task are listed in Table 3. In the oxygen radical absorption capacity (ORAC), singlet oxygen absorption capacity (SOAC), and DPPH tasks, no chemical inconsistencies were observed in the interpretation of supervised learning models. Additionally, in ORAC, topological polar surface area (TPSA), which counts the oxygen atoms in the OH group, was detected as an important feature, while IP was identified as an important feature of SOAC. The energy of the highest occupied molecular orbital (HOMO) (*E*_HOMO_) was previously shown to play a role in the scavenging of singlet oxygen, in both energy and electron transfer [30]. It is also known that IP and *E*_HOMO_ are inversely correlated [31]. Considering that *E*_HOMO_ was removed from the explanatory variables by feature selection, it is reasonable to assume that IP dominated the predictive trend instead of *E*_HOMO_. As for DPPH, the TPSA, BalabanJ, and HOMO–LUMO gap were identified as key features, and these are thought to indicate the size of the electron-rich area, steric hindrance, and ease of electron transfer, respectively.

To examine the differences in the predictive performance of the model with changes in the number of features, we prepared two types of pipelines for each task. One of these was a linear model trained with different features, whilst the other was a comparative XGBoost model trained with a larger number of features. These models are summarized in Table 4 and Table 5, respectively. A linear model, which is an ordinary least squares linear regression (OLS) model, was used for regression tasks, whereas a logistic regression model was used for classification. Table 4 shows that the XGBoost models trained using 18 common features performed better than the linear models. This suggests that it was difficult to describe antioxidant activity using only a few features. The features used for training the comparative XGBoost models included these 18 common features and discrete distributions, such as PEOE_VSA. These were filtered using the variance of each feature and the correlation coefficients of the feature pairs. Table 5 shows that 18 features were sufficient in explaining the trends of ORAC, SOAC, and DPPH. However, for the MTT task, the predictive performance did not change when the number of features increased. It was found that the features were reduced, whilst retaining essential information to explain the antioxidants. The above results show that 18 features are sufficient to explain the relationship between molecular information and multiple antioxidant capacity indices. Although predictions have been made for individual indicators in the past, the presentation of features that can comprehensively explain the behavior of multiple indicators is expected to promote the compound screening process. Furthermore, both properties obtained by computational chemistry and geometrical molecular structure information were found to be necessary to predict activity.

### 2.3. Chemical Space in Uniform Manifold Approximation and Projection (UMAP)

**Figure 4:**
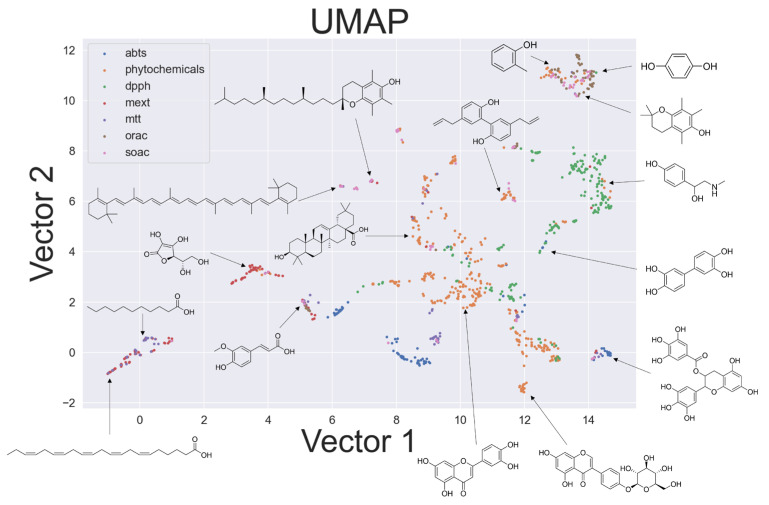
Distribution of compounds related to antioxidant capacity and assay produced by UMAP.

The two-dimensional distribution of the compound output by UMAP [32] is shown in Figure 4. When explaining multiple activities with small similarities, a two-dimensional representation is easier to describe and is more readable. Therefore, we generated a two-dimensional distribution. UMAP has become popular in recent years as a means of dimensionality reduction and data visualization owing to its high processing speed and clear separation of data clusters. UMAP generates graphs in low-dimensional (two-dimensional in this paper) conditions that are similar to graphs produced with higher-dimensional (18-dimensional) information. Data with similar values are clustered in neighborhoods, while pairs with large differences are distributed apart. As a result of UMAP, compounds with similar structures formed groups. Focusing on the position of the distribution, phenols, glycosides, fatty acids, and carotenoids were present radially, with flavonoids at the center. This radial distribution could be attributed to differences in the ROS preferentially scavenged by each compound group, reaction mechanisms, and solubility. The qualitative estimation of the activity of a certain compound against different ROS is expected according to the structure and calculated values, along with the suggestion of an appropriate assay. This will enable rapid analysis of antioxidant capacity covering multiple reactions of natural products and new compounds. Furthermore, observing the structures of the compounds corresponding to some of the plots, a trend was observed on the axes in Figure 4. The horizontal axis in Figure 4 provides information on the number and polarity of the hydroxy groups in the molecule, whereas the vertical axis indicates information on the surface area and bulkiness of the three-dimensional structure of the molecule. However, when outputting the two-dimensional distribution in this study, the axes were not defined in advance. Therefore, TPSA for polarity, MolLogP for conformation, LabuteASA, and FractionCSP3 for steric structures were all particularly important when describing the overall picture of antioxidants. The distribution of these data obtained from 200 molecular descriptors, ECFPs, and 15 features is shown in Supplementary Figures S13–S15; Figures S13 and S14 were generated in ChemPlot. The features used for the output of Supplementary Figure S15 are the common features, excluding the BDE, IP, proton affinity (PA), and other properties obtained from the PM7 calculations. Compared to Supplementary Figures S13–S15, Figure 4 shows that outliers and agglomerations do not occur in most data, and the chemical space is represented. This suggests that the feature selection in this study was effective in describing the chemical space of antioxidants.

## 3. Materials and Methods

### 3.1. Data Collection

The tasks to be solved using the supervised learning models are listed in Table 1. Four regression tasks and a classification task were prepared for antioxidant assays. Each task contained a small dataset, and a total of 104 numeric features were obtained from RDKit (Open-source cheminformatics. https://www.rdkit.org accessed on 6 December 2022). Nine features were then calculated using a semi-empirical method (PM7) with MOPAC2016 [33]. Density functional theory (DFT) calculations using B3LYP or M06-2X functionals have been commonly used to determine the reactivity of molecules [34]. However, Nakata et al. [35] showed a similar behavior between the energies of the frontier orbitals of 2.6 million molecules calculated using PM6 (the predecessor method of PM7) and those calculated by B3LYP/6-31G*. Considering the accuracy and cost of calculations, it was reasonable to use PM7 here instead of DFT. The energies at the HOMO, the LUMO, the HOMO–LUMO gap, and the dipole moment of each compound were all calculated from the optimized structure of the neutral molecules. Thermodynamic properties, such as BDE, IP, proton dissociation enthalpy (PDE), PA, and electron-transfer enthalpy (ETE), were then also calculated using the formation energies of neutral molecules, hydrogen-withdrawn radicals, radical cations, and anions with Equations (1)–(5). 

**Figure 5:**
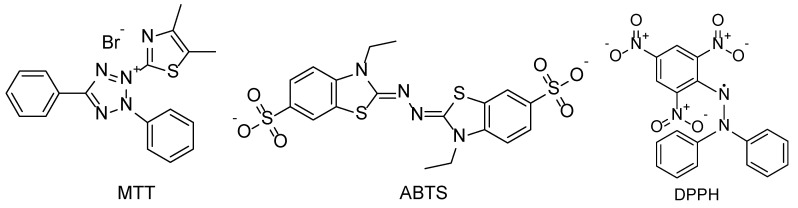
Structures of radicals used in antioxidant assays.

Each of the datasets used was collected from various past studies, as shown in the dataset column of Table 1. ORAC, SOAC, MTT, ABTS, and DPPH assays were used to measure the antioxidant properties in diverse ways, as the radicals that reacted with antioxidant samples were different in each of these assays. For example, ROS were used in the first two assays, whilst the radicals shown in Figure 5 were used in the latter three cases. The ORAC dataset contained 70 phenolics [3], and the ORAC assay is a method that monitors differences in the fluorescence change of probe molecules over time at certain wavelengths as a result of sample addition [36,37]. Since antioxidants prevent peroxyl radicals from attacking fluorescence probe molecules, samples with high ORAC values exhibit a slower fluorescence decay. ORAC values were determined relative to Trolox, 6-methoxy-2,5,7,8-tetramethylchromane-2-carboxylic acid. The SOAC dataset contained 71 compounds, such as phenolics and carotenoids [4]. The SOAC assay is a method used for measuring the reaction rate between singlet oxygen and samples [38], and, in this case, SOAC values were determined relative to those of α-tocopherol. Additionally, the MTT dataset contained 71 compounds, which included phenolics and fatty acids [20], whilst the MTT assay was performed using the substrate 3-(4,5-dimethylthiazole-2-yl)-2,5-diphenyltetrazolium bromide. Considering that the color of MTT changes from yellow to purple through reduction, the absorbance of the reaction mixtures with samples at 570 nm was used as the MTT value. Furthermore, the ABTS dataset contained 90 phenolic compounds from Chinese medicinal plants [21], and the ABTS assay used a reduction reaction of the 2,2′-azinobis(3-ethylbenzothiazoline-6-sulfonic acid) radical cation [39]. The absorbance of the reaction mixtures with the samples at 734 nm indicated the concentration of radical cations. ABTS values were then expressed as Trolox equivalent antioxidant capacity (TEAC) using the half-maximal inhibitory concentration (IC_50_) obtained from absorbance. TEAC was calculated using the IC_50_ and Equation (6).

(6) $$\mathrm{TEAC}=\frac{{\mathrm{IC}}_{50}\;\mathrm{of}\;\mathrm{Trolox}\;\left[\mu M\right]}{{\mathrm{IC}}_{50}\;\mathrm{of}\;\mathrm{samples}\;\left[\mu M\right]}.$$

Lastly, the DPPH dataset contained 198 phenolic compounds [12], whilst the DPPH assay measured the difference in the concentration of DPPH in the reduction reaction from absorbance [40], similarly to the ABTS assay. To make the DPPH dataset available for classification, compounds with IC_50_ below 300 μM were considered positive, whilst the remainder were considered negative. The DPPH dataset was composed of 97 positive and 101 negative samples.

Two large datasets without target values for prediction were also prepared to describe the chemical space of the antioxidants. One dataset contained information on phytochemicals [26] sold by Tokyo Chemical Industry Co., Ltd (Tokyo, Japan). The other included compounds written in the Standard Tables of Food Composition in Japan (Seventh Revised Edition) [27] published by the Ministry of Education, Culture, Sports, Science, and Technology (Tokyo, Japan). For these two datasets, common features were prepared alongside the other five datasets and were selected using the method described in the next section. All compounds used in this study are shown as compounds_data.zip in the Supplementary Materials.

### 3.2. Feature Selection

**Scheme 1:**
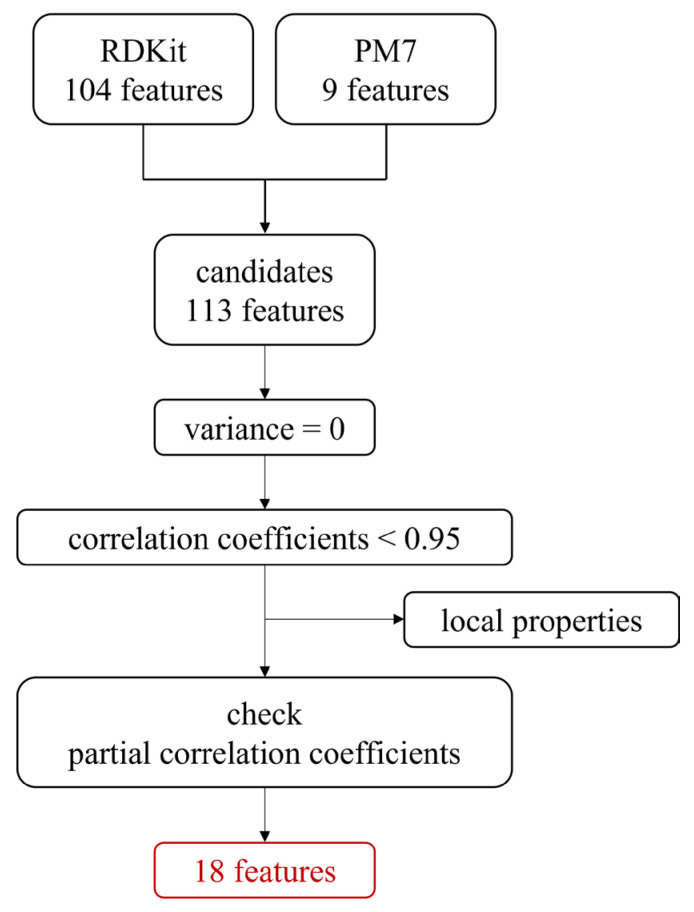
The method of feature selection. There were four steps for reducing features. Variance and correlation coefficients were used as the threshold values in the first two steps. Then, in the third step, discrete features, or local properties in a molecule, were dropped. Partial correlation coefficients were subsequently used as the threshold values in the last step.

Feature selection was performed using the filter method, and the feature selection scheme is shown in Scheme 1. Firstly, the constant features were dropped, and multicollinearity was removed by considering correlation coefficients. The threshold for the absolute value of the correlation coefficient was 0.95. Features representing the local properties within the molecule, such as PEOE_VSA and SlogP_VSA, were also removed. Most of these descriptors exhibited discrete behavior since they referred to the surface of a functional group. To describe molecules with a small number of features, continuous values determined by a single value per molecule were used here. Furthermore, to explain the relationship of the features correctly, they were screened on the basis of partial correlation coefficients. This sequence of operations was repeated for each dataset to select 18 common features available for the prediction of multiple antioxidant capacities.

### 3.3. Supervised Learning

The datasets corresponding to each task were then inputted into an XGBoost model. The machine learning models used here were screened in advance using EvalML, an automated machine learning library (Alteryx, Inc., Irvine, CA, USA). XGBoost was used for supervised learning in this study considering that it showed a stable performance for the prediction of five tasks. The parameters of XGBoost were optimized for each dataset using Optuna [41], a hyperparameter optimization framework. The MAE was used as the loss function for regression tasks, whilst the RMSE was also calculated for evaluation. These metrics were obtained using Equations (7) and (8), where y and $\hat{y}$ are the target value in the dataset and its prediction, respectively. MAE/STD was prepared to compare errors among the machine learning models trained using different datasets. The MAE was then divided by the standard deviation of the target values in each dataset. For a classification task, the binary cross-entropy shown in Equation (9) was used as a loss function, where $y\in \left\{0,\;1\right\}$ is a target value in the dataset. P means the probability that y=1. Accuracy was also prepared for the evaluation metrics from Equation (10), where TP, FP, TN, and FN refer to the numbers of true positive, false positive, true negative, and false negative data, respectively.

(7) $$\mathrm{MAE}=\frac{\sum_{i=0}^{n-1}\left|y-\hat{y}\right|}{n}.$$

(8) $$\mathrm{RMSE}=\sqrt{\frac{\sum_{i=0}^{n-1}{\left(y-\hat{y}\right)}^{2}}{n}}.$$

(9) $$\mathrm{Binary}\;\mathrm{cross}\text{-}\mathrm{entropy}=-\left\{y\log P+\left(1-y\right)\log\left(1-P\right)\right\}.$$

(10) $$\mathrm{Accuracy}=\frac{\mathrm{TP}+\mathrm{TN}}{\mathrm{TP}+\mathrm{TN}+\mathrm{FP}+\mathrm{FN}}.$$

XGBoost was useful not only for effective predictions but also for gaining insight into features. Feature importance was obtained from the trained XGBoost model for each task, although, in general, these values were not quantitatively comparable when obtained from different models. To understand the qualitative trends, the top features were sorted and analyzed in order of importance as described by Funatsu et al. [42] Furthermore, to describe the difference in the importance of the two features, the feature importance values (x) were scaled into a range between 0 and 1 according to Equation (11). The value with the highest contribution to the prediction was transformed to 1, whereas the value with the lowest contribution was transformed to 0. Features with scaled importance greater than 0.85 were considered key features in each task.

(11) $$x_{\mathrm{scaled}}=\frac{x-x_{\min}}{x_{\max}-x_{\min}}.$$

### 3.4. Dimension Reduction

A UMAP was used to visualize the chemical space, and the set parameters of this UMAP are shown in the Supplementary Materials. Data from the seven datasets listed in Table 1 were input into the UMAP simultaneously. Subsequently, the UMAP compressed these data into two dimensions. The target values were then removed, and 18 common features were standardized according to Equation (12) in advance, where μ is the mean and σ is the standard deviation of feature x.

(12) $$x_{\mathrm{std}}=\frac{x-\mu }{\sigma }.$$

## 4. Conclusions

For the comprehensive analysis of antioxidant mechanisms, several datasets related to the antioxidant capacity test were analyzed here. For this, 18 features were selected as information that could describe the antioxidant mechanism using both domain knowledge and statistical processing. Through supervised learning, it was confirmed possible to estimate antioxidant capacity using a small amount of data containing the selected features. Dimension reduction was also used to comprehensively describe antioxidant activities as a chemical space and to visualize the importance of polarity and conformation, reflecting trends in compound structure and activity. Considering that scavenging mechanisms of ROS have been proposed, here, we added descriptors obtained from quantum chemical calculations. As a result, both structural information and the reactivity of molecules were applied in this study for the evaluation of antioxidant capacity. It can be concluded that comprehensive antioxidant capacity analysis is possible by using both geometric information and computational chemistry, and it is expected to facilitate data analysis of biological activity and physical properties of organic compounds and to recommend appropriate assays.

In the future, we would like to include data from other antioxidant assays to improve the generalizability of the analysis methods in this study. As we now know the importance of polarity and 3D structure, we would like to create a general-purpose antioxidant capacity index based on this information.

## Figures and Tables

**Table 2:** Selected 18 common features in this study. Features in the energy class and dipole moment were calculated with PM7. Other features were obtained from RDKit.

Class	Number	Features	Explanation
Energy	5	*E*_LUMO_	Energy of LUMO
		gap	HOMO–LUMO gap
		BDE	Bond dissociation energy
		IP	Ionization potential
		PA	Proton affinity
Polarity	5	TPSA	Topological polar surface area estimated from bonding patterns
		MolLogP	Octanol–water partition coefficients estimated from fragments
		dipole moment	The dipole moment calculated with PM7
		MaxPartialCharge, MinPartialCharge	Maximum and minimum values of atomic charges in a molecule
Structure and bonds	6	FpDensityMorgan2	Possible substructure variations
		BertzCT	Molecular complexity caused by the kinds of atoms and the bond order
		BalabanJ	Topological index based on the distance matrix of a molecule
		HallKierAlpha	Sum of the relative covalent radius in a molecule
		MaxEStateIndex, MinEStateIndex	Maximum and minimum values determined for each fragment
Steric properties	2	LabuteASA	Molecular surface area where solvents are accessible
		FractionCSP3	Ratio of sp^3^ carbons among the carbons in the molecule

**Table 3:** Evaluation of the performance of XGBoost models for prediction of test data properties. Lower MAE, RMSE, and cross-entropy are better. Higher accuracy shows a better performance. MAE/STD is the MAE divided by the standard deviation of target values in each dataset. The higher importance column lists features with importance greater than 0.85 after scaling.

**Regression**				
**Dataset**	**MAE**	**RMSE**	**MAE/STD**	**Higher Importance**
ORAC	0.5314	0.7295	0.2724	TPSA
SOAC	1.4555	1.9020	0.3684	IP
MTT	0.8000	1.0420	0.5774	BDE
ABTS	0.1559	0.1939	0.6476	HallKierAlpha
**Classification**				
**Dataset**	**Cross-entropy**	**Accuracy**		**Higher importance**
DPPH	2.8783	0.9167		TPSA, BalabanJ, gap

**Table 4:** Comparison of XGBoost and linear models based on predictive performance for test data. Ordinary least squares linear regression (OLS) models and a logistic regression model were trained with different features. MAE_18_ means the MAE values of a model trained with 18 common features. MAE_linear_ means the MAE of a model trained with input features shown in this table. Accuracy was used in the DPPH task instead of MAE. In most cases, models trained using 18 common features showed a better performance. * better performance values.

**Regression**				
**Task**	**Model**	**Input Features**	**MAE_18_**	**MAE_linear_**
ORAC	OLS	BDE, TPSA	0.5314 *	1.2720
SOAC	OLS	IP	1.4555 *	2.0491
MTT	OLS	IP	0.1559 *	0.2091
ABTS	OLS	BDE, IP	0.8000	0.6833 *
**Classification**				
**Task**	**Model**	**Input features**	**Accuracy_18_**	**Accuracy_linear_**
DPPH	Logistic	Gap, IP, TPSA, BalabanJ	0.9167 *	0.9000

**Table 5:** Comparison of XGBoost models based on predictive performance for test data. The column “Number of features” shows the number of input features of comparative XGBoost models. MAE_18_ means the MAE of a model trained with 18 common features, whereas MAE_complex_ is the MAE of a model trained with a larger number of features. Accuracy was used in the DPPH task instead of MAE. * better performance values.

**Regression**			
**Task**	**Number of Features**	**MAE_18_**	**MAE_complex_**
ORAC	54	0.5314 *	0.5900
SOAC	65	1.4555 *	1.5684
MTT	61	0.1559	0.1544 *
ABTS	62	0.8000	0.6157 *
**Classification**			
**Task**	**Number of features**	**Accuracy18**	**Accuracycomplex**
DPPH	74	0.9167	0.9500 *

## Data Availability

All data are contained in the article and Supplementary Materials.

## Supplementary Materials

The following supporting information can be downloaded at https://www.mdpi.com/article/10.3390/molecules28031454/s1: Figures S1–S7. Scatter plot matrices of all datasets used in this study; Figures S8–S12. Partial correlation matrices of datasets used in supervised learning; Figures S13–S15. UMAP plots obtained from 200 molecular descriptors, ECFP, and 15 features; feature_importance.xlsx: Feature importance of XGBoost models for each task; compounds_data.zip: All data on compounds used in this study. These were prepared in CSV format.
