# Supplementary material for: Feature Selection for the Interpretation of Antioxidant Mechanisms in Plant Phenolics
Source: Molecules. 2023 Feb 2;28(3):1454. doi: 10.3390/molecules28031454 (PMC9921549; doi:10.3390/molecules28031454)
Supplement: Supplementary file 1 [file molecules-28-01454-s001.zip › molecules-2142870-supplementary.pdf]

Molecules

The Supplementary Materials of

Feature selection for comprehensive interpretation of antioxidant mechanisms in Plant Phenolics

Taiki Fujimoto and Hiroaki Gotoh

Scatter plot matrices

The scatter plot matrices of datasets used in this study were shown below. Diagonal components give the distribution of each feature. Other components give a correlation between the two features.

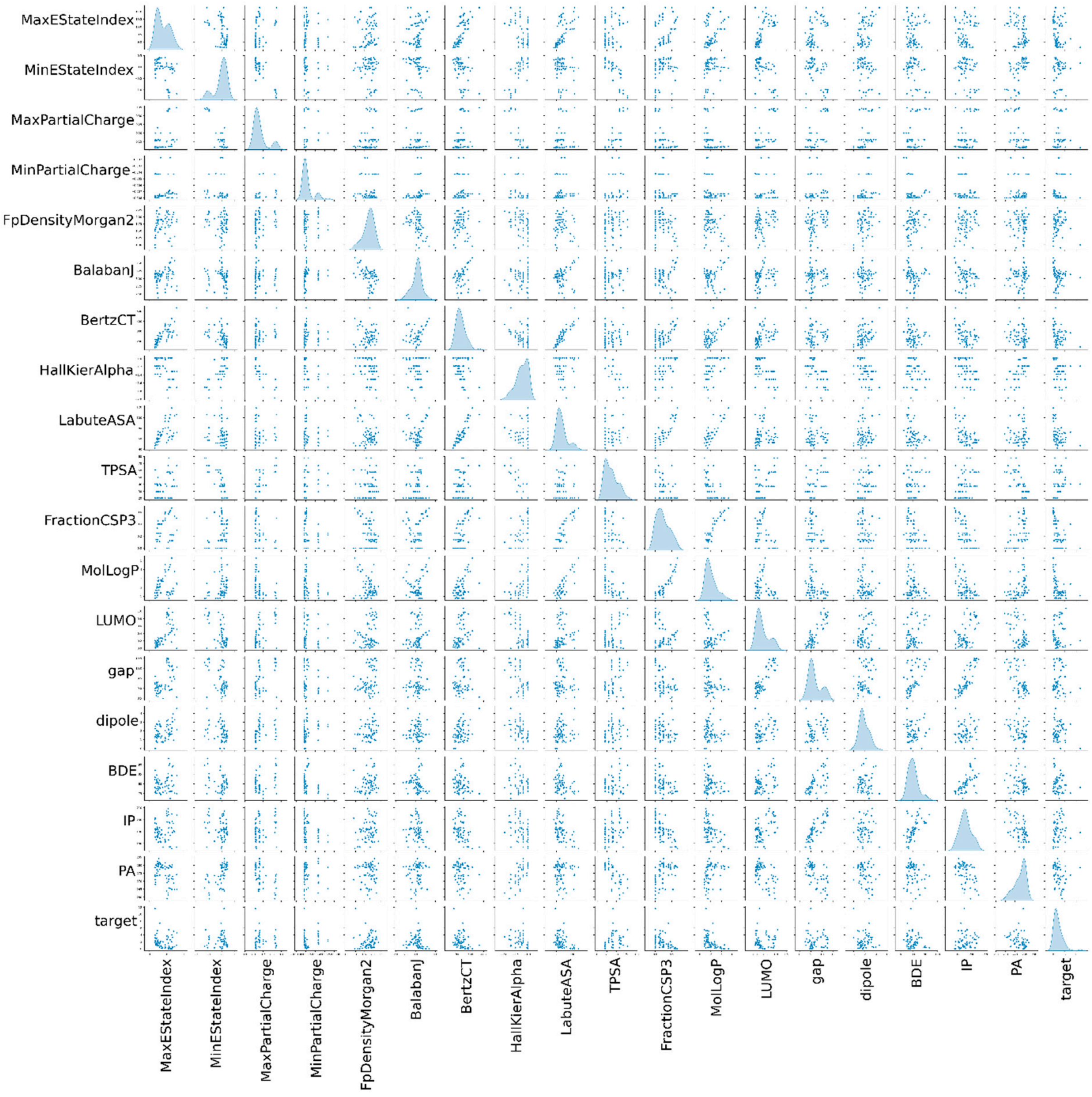

Figure S1 The scatter plot matrix of the ORAC dataset [1].

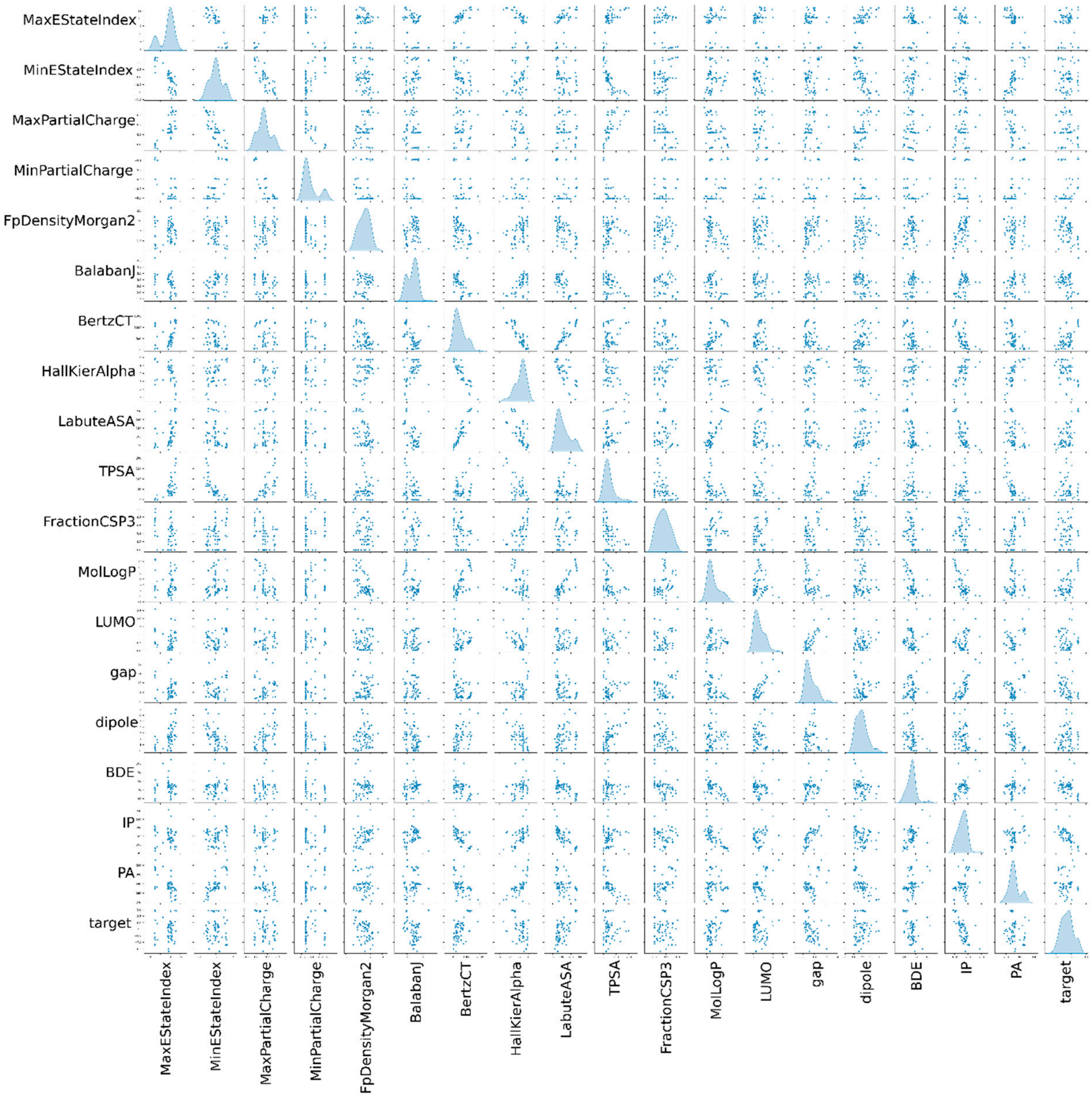

Figure S2 The scatter plot matrix of the SOAC dataset [2].

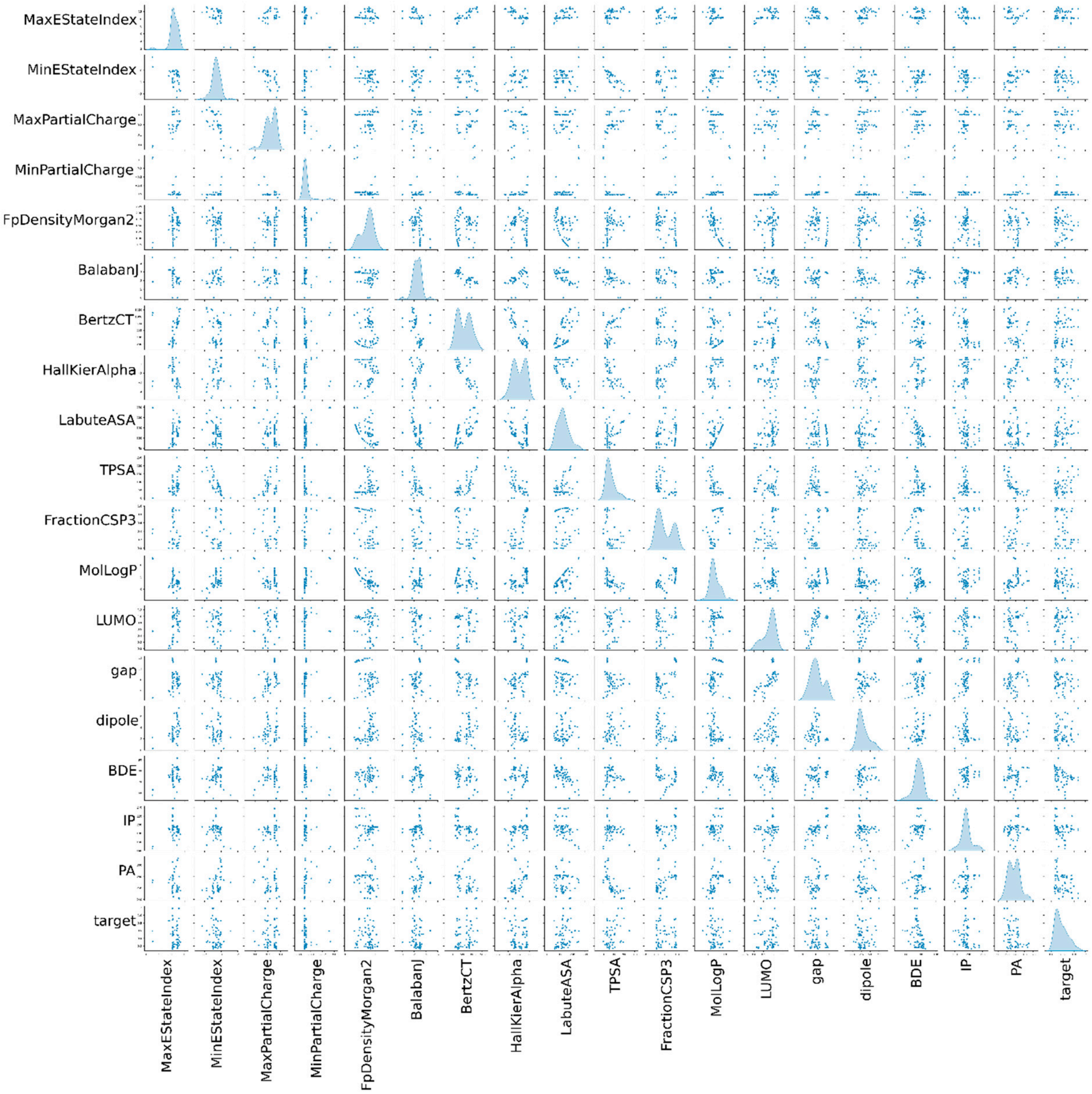

Figure S3 The scatter plot matrix of the MTT dataset [3].

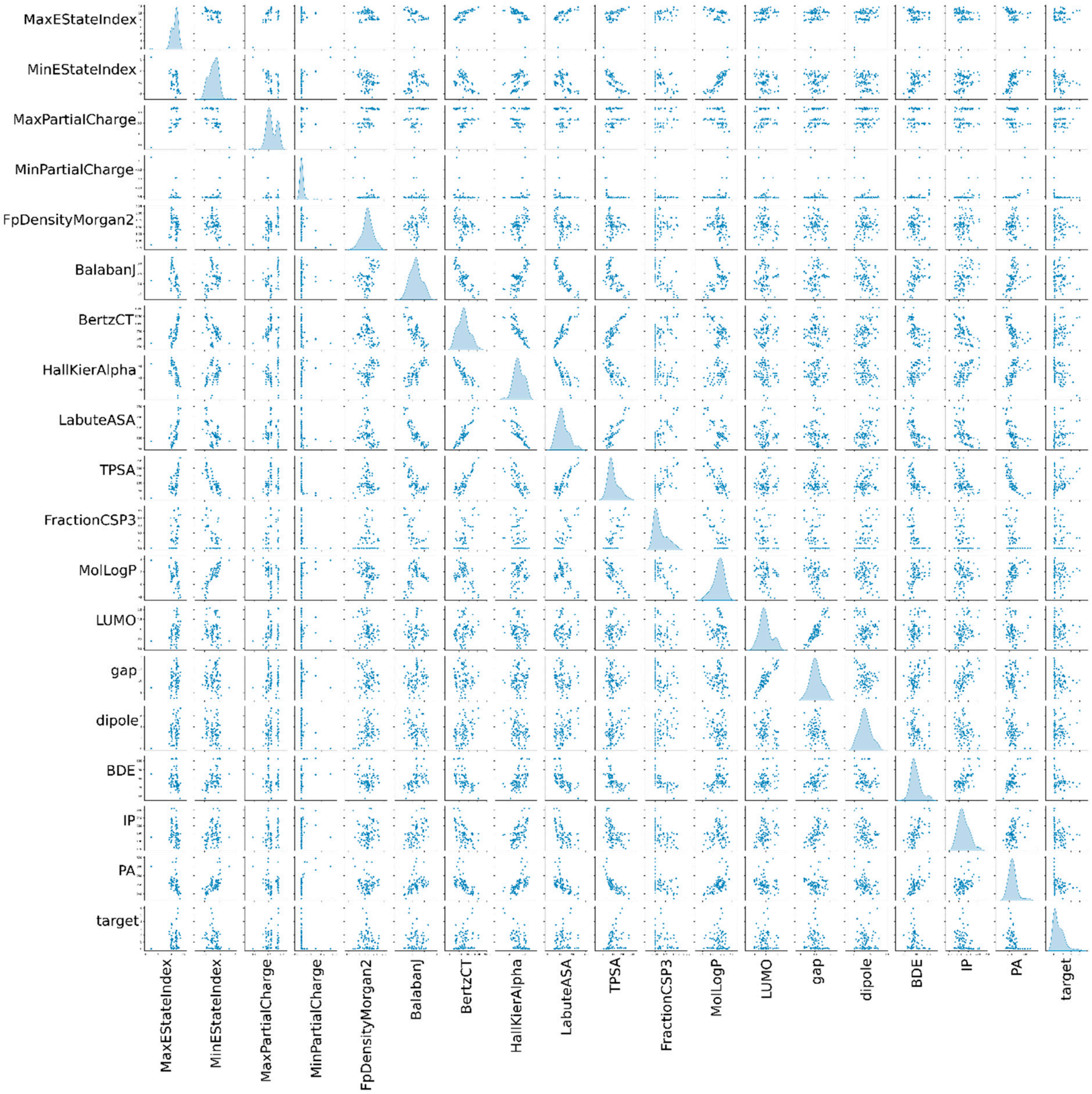

Figure S4 The scatter plot matrix of the ABTS dataset [4].

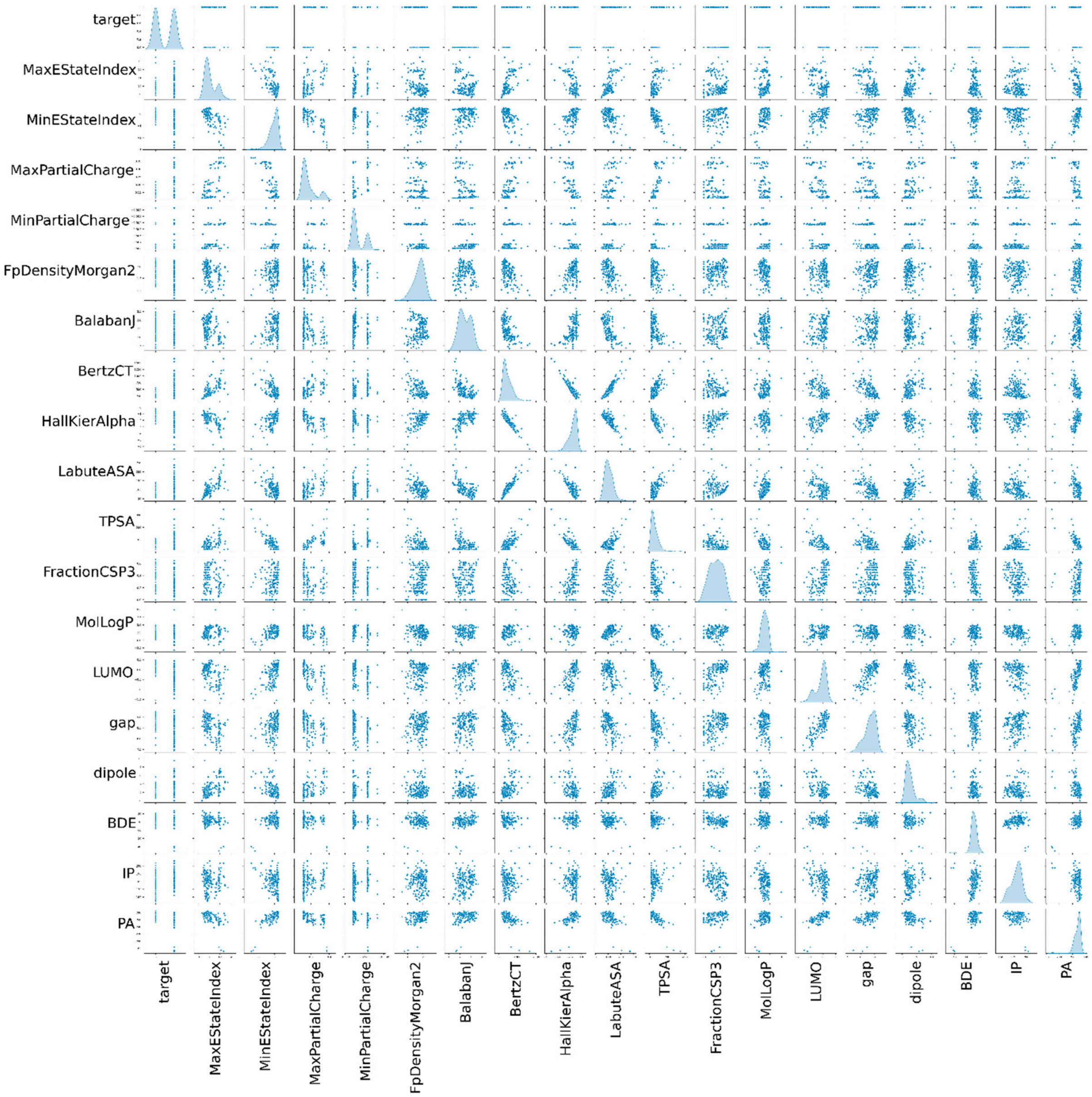

Figure S5 The scatter plot matrix of the DPPH dataset [5]. Since DPPH dataset was used to solve a classification task, target values were dropped at this figure. This dataset is composed of 97 positive data and 101 negative data.

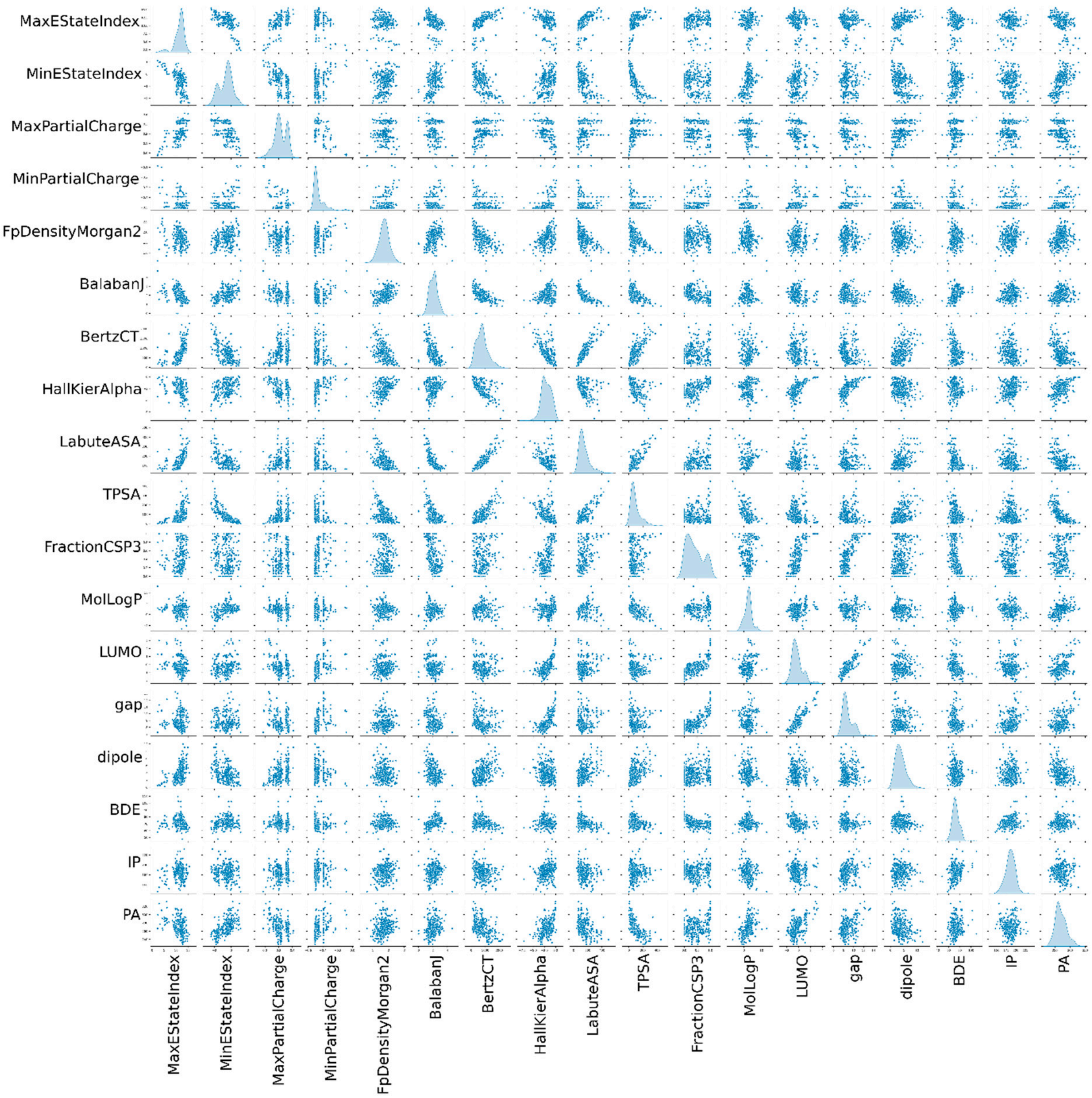

Figure S6 The scatter plot matrix of the dataset of phytochemicals sold by Tokyo Chemical Industry Co., Ltd. [6]

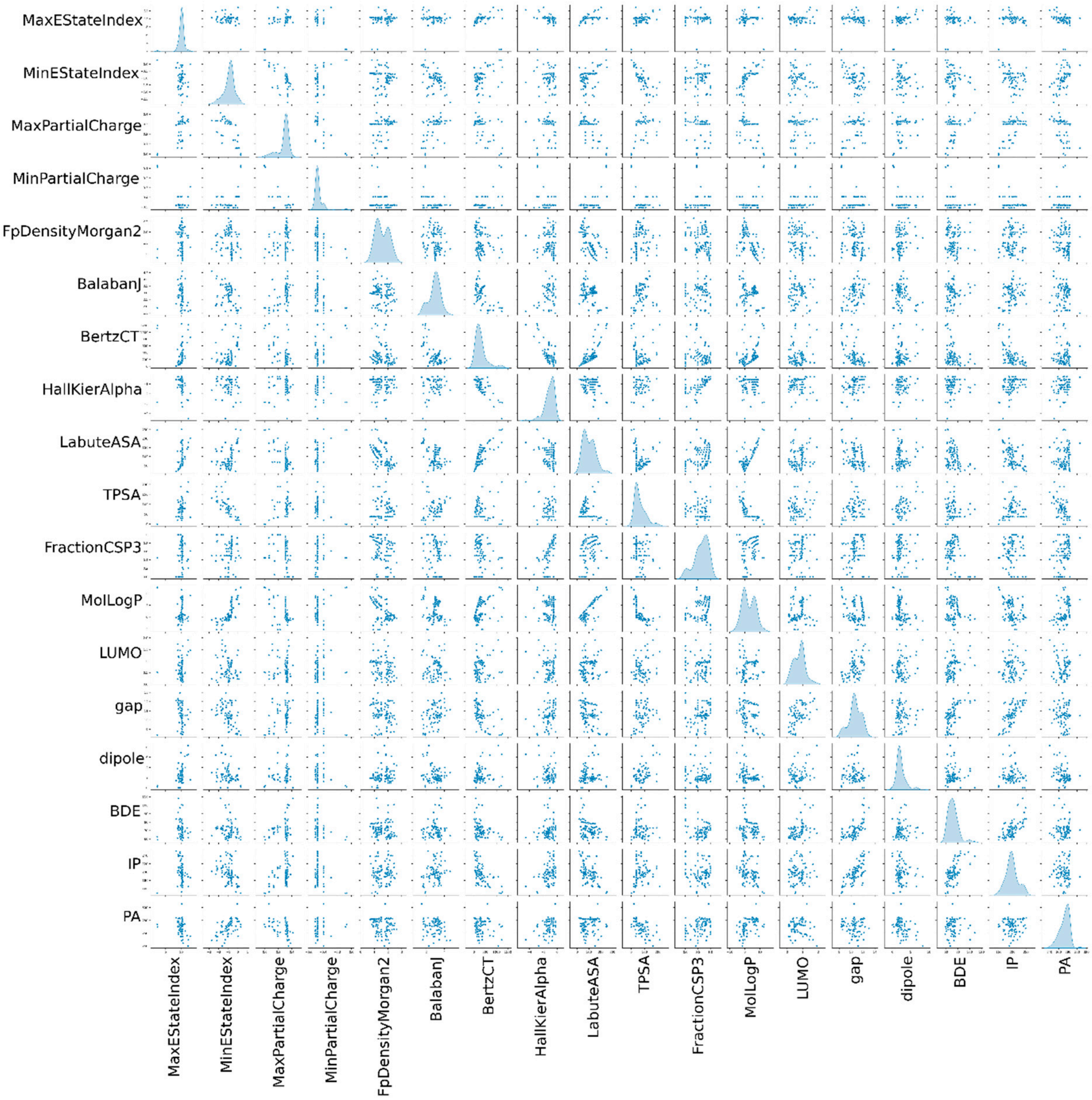

Figure S7 The scatter plot matrix of the dataset of compounds on Standard Tables of Food Composition in Japan [7].

Partial correlation matrices

Partial correlation matrices obtained from datasets used in the supervised learning were shown below as heatmaps. The partial correlation matrix ρ is obtained from the inverse of the variance-covariance matrix Π^{-1} as shown in Equation S1. Each component ρ_{ij} represents the correlation between residuals obtained by sub-tracting the contributions of other features from the corresponding features. In this way, we confirmed that no dependency existed between the features.

$$\rho_{\mathrm{ij}} = -\frac{p_{\mathrm{ij}}}{\sqrt{p_{\mathrm{ii}}p_{\mathrm{jj}}}} \quad \text{when } \Pi^{-1} = \begin{pmatrix} p_{11} & \cdots & p_{1\mathrm{n}} \\ \vdots & \ddots & \vdots \\ p_{\mathrm{n}1} & \cdots & p_{\mathrm{nn}} \end{pmatrix} \quad \text{(S1)}$$

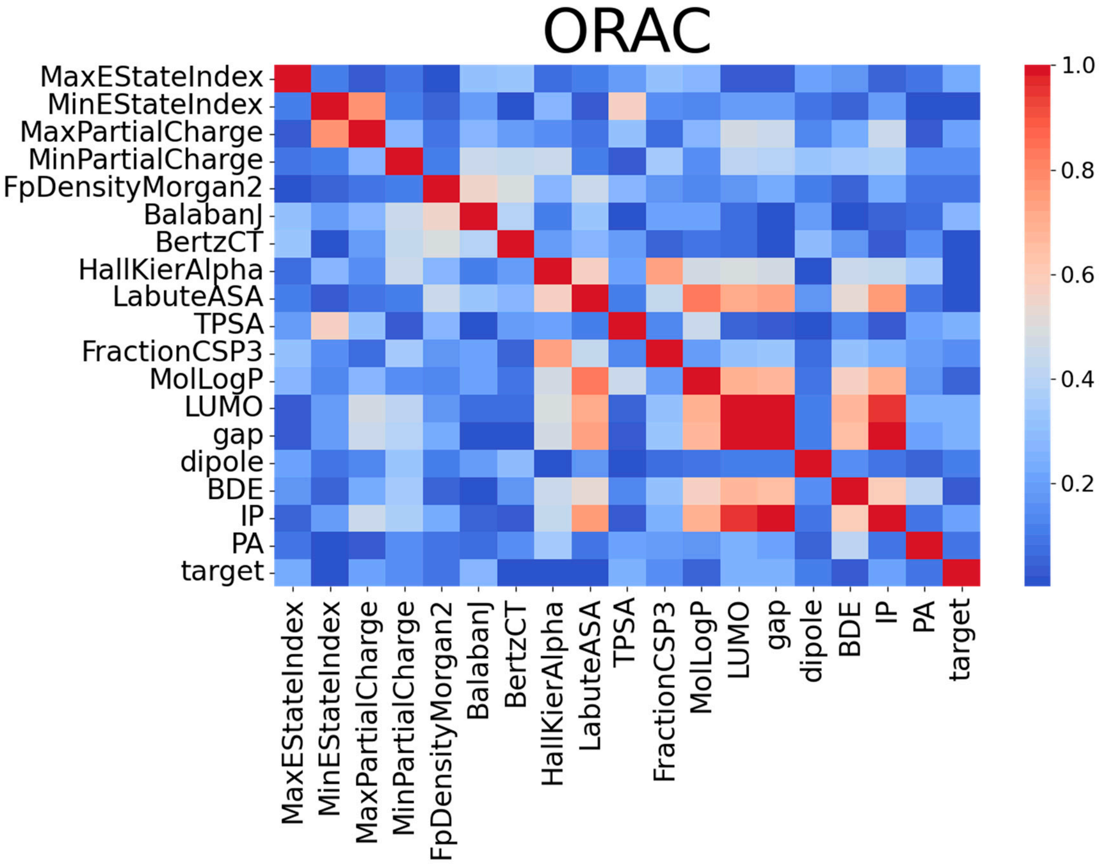

Figure S8 The partial correlation matrix of the ORAC dataset.

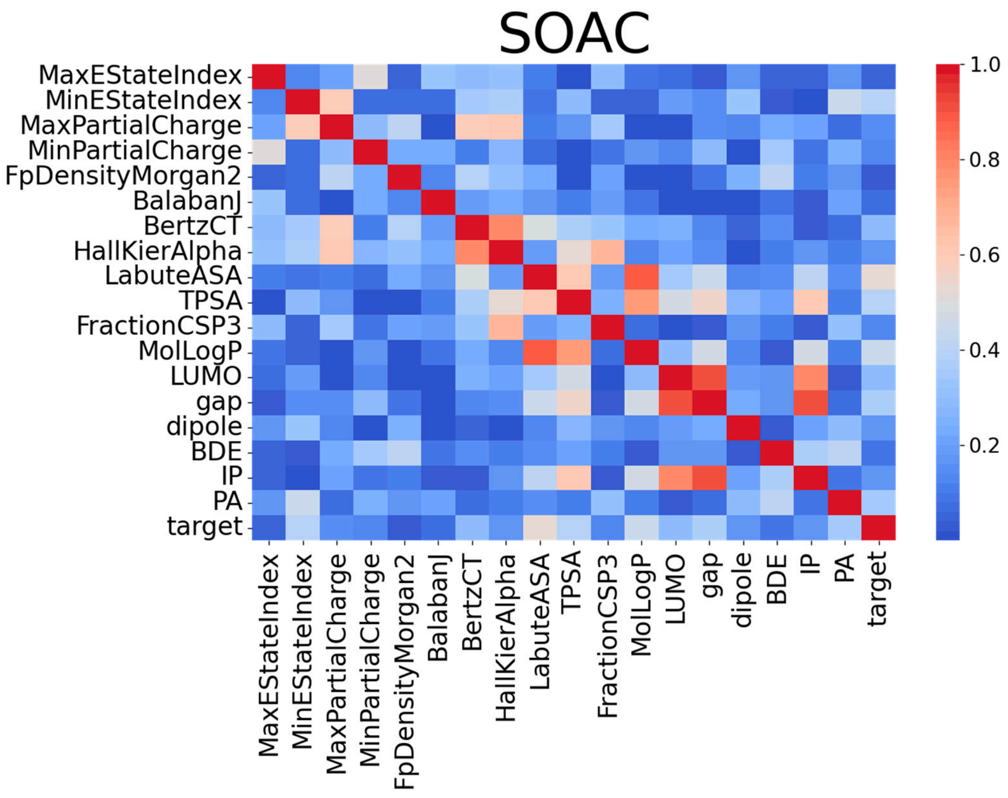

Figure S9 The partial correlation matrix of the SOAC dataset.

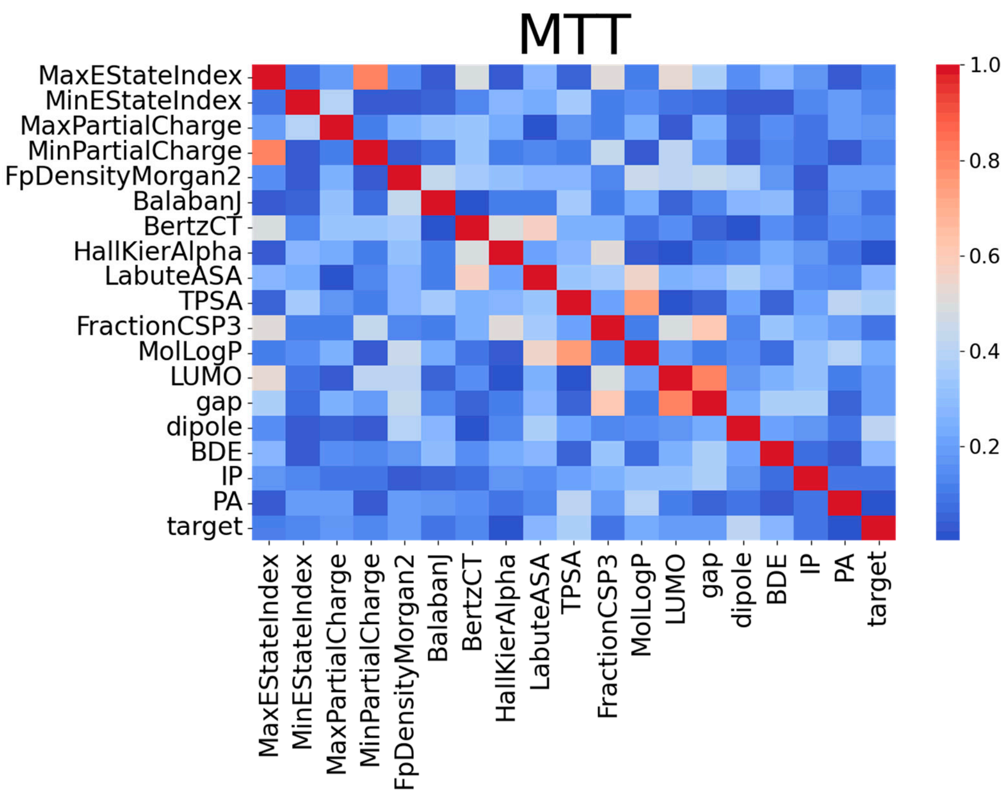

Figure S10 The partial correlation matrix of the MTT dataset.

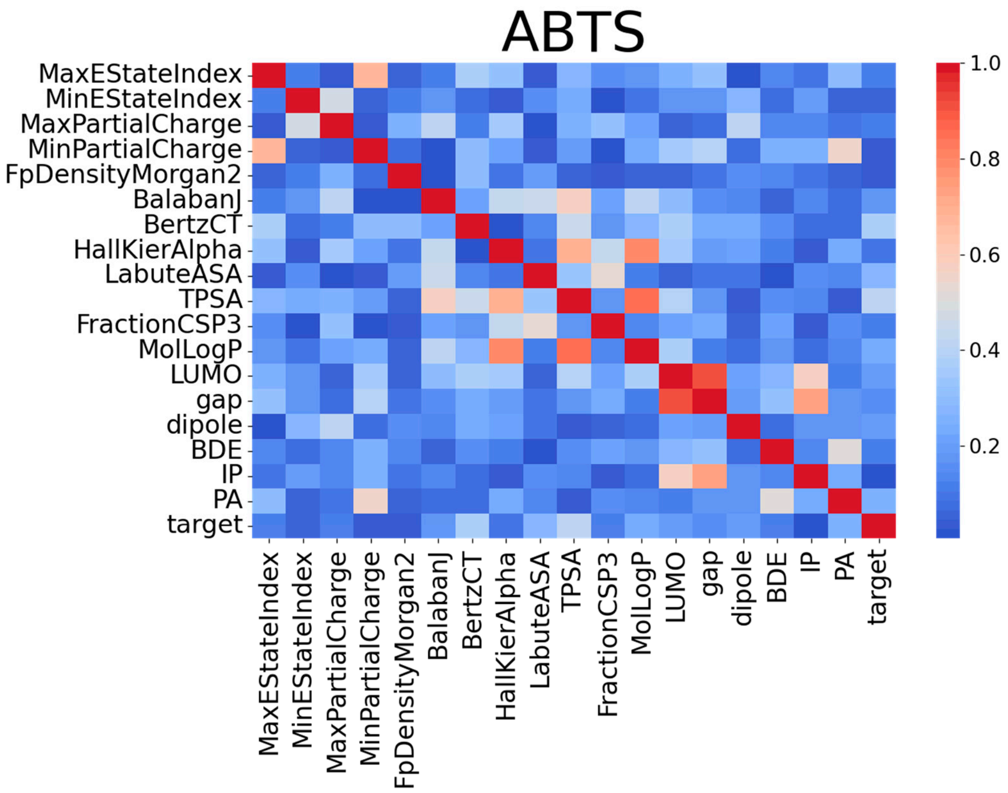

Figure S11 The partial correlation matrix of the ABTS dataset.

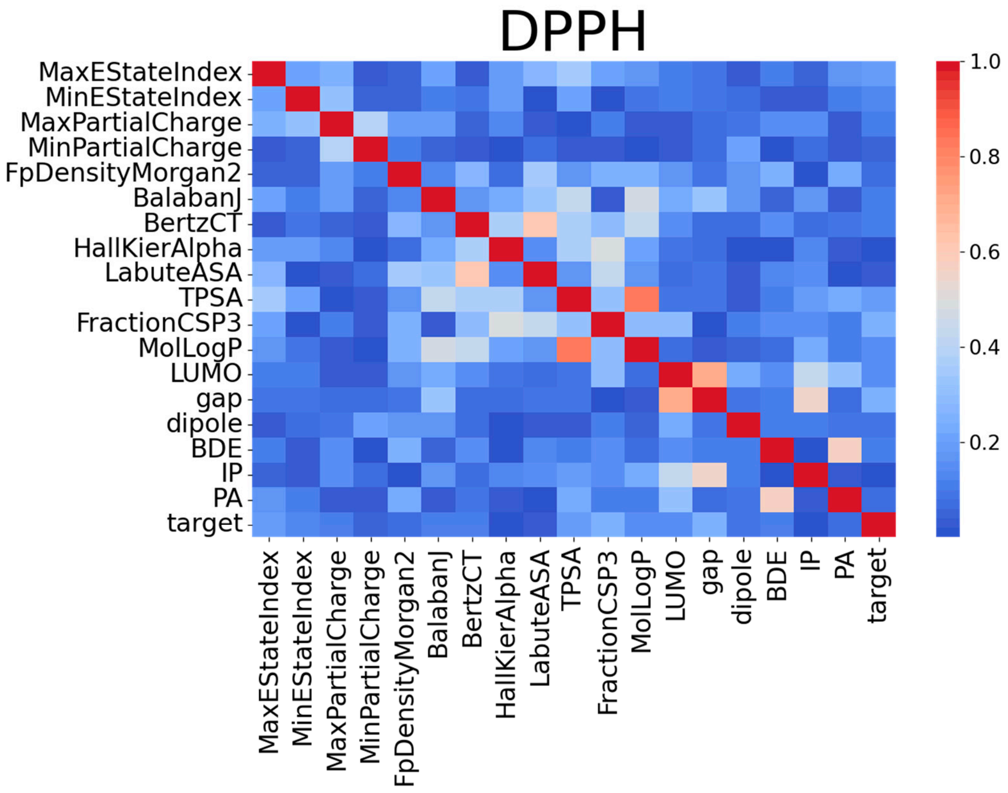

Figure S12 The partial correlation matrix of the DPPH dataset.

Comparison of UMAP

The two UMAP [8] parameters in this study, n_neighbors, and min_dist were uniformly set to 15 and 0.1, respectively. The former is a parameter that adjusts the ease of plot aggregation, and the latter is a parameter that sets the minimum distance between each plot. UMAP plots obtained from 200 molecular descriptors, extended connectivity fingerprints [9], and 15 features were shown in Figure S13-15, respectively. In Figure S13 and S14, since most compounds were clustered at the center, it is difficult to make a chemical interpretation from these two figures. Figure S15 was obtained by using 15 features excluding features calculated with PM7 such as bond dissociation energy (BDE), ionization potential (IP), and proton affinity (PA).

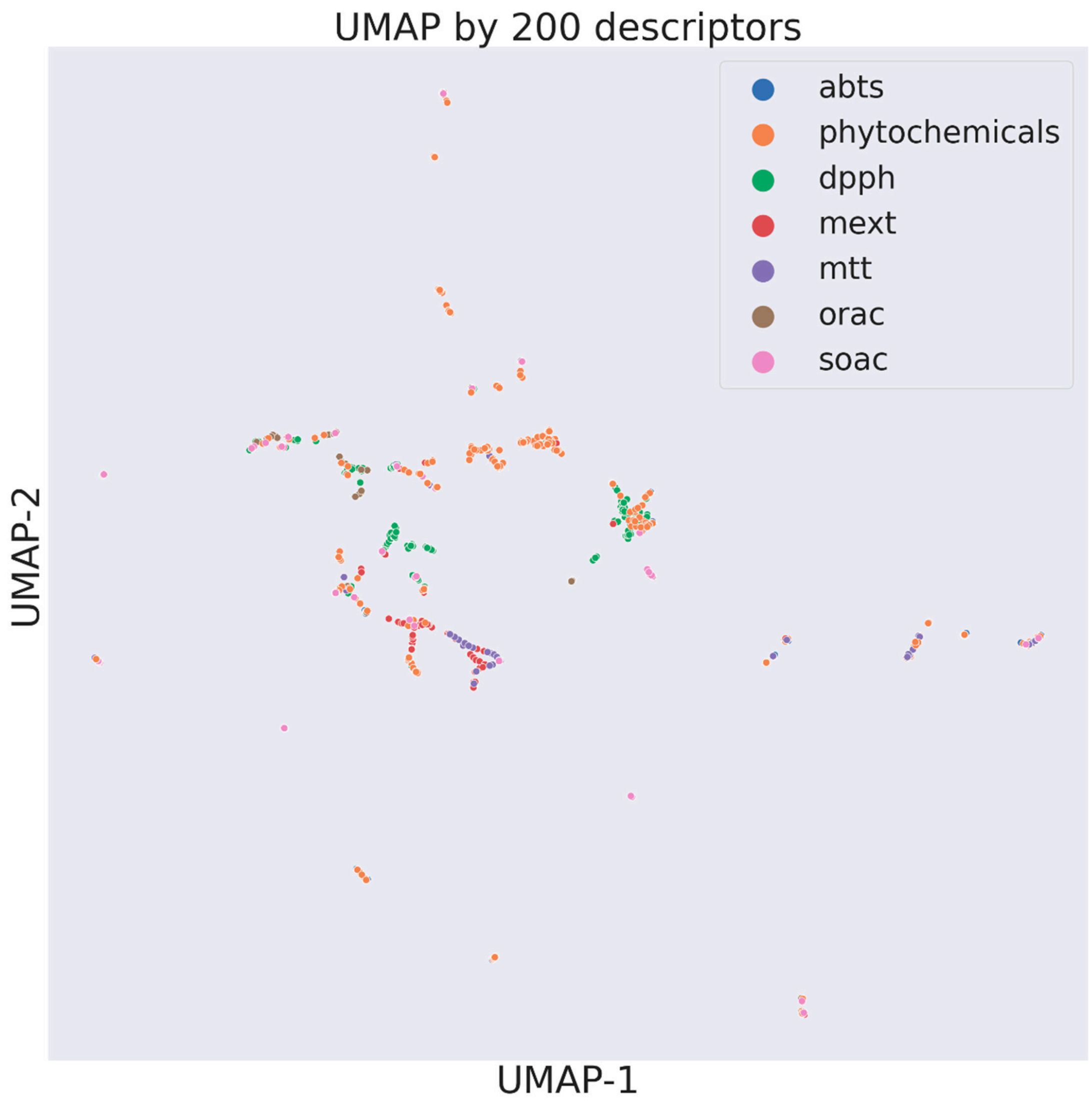

Figure S13 Distribution of antioxidants obtained from 200 molecular descriptors by using UMAP. It is generated by ChemPlot [10].

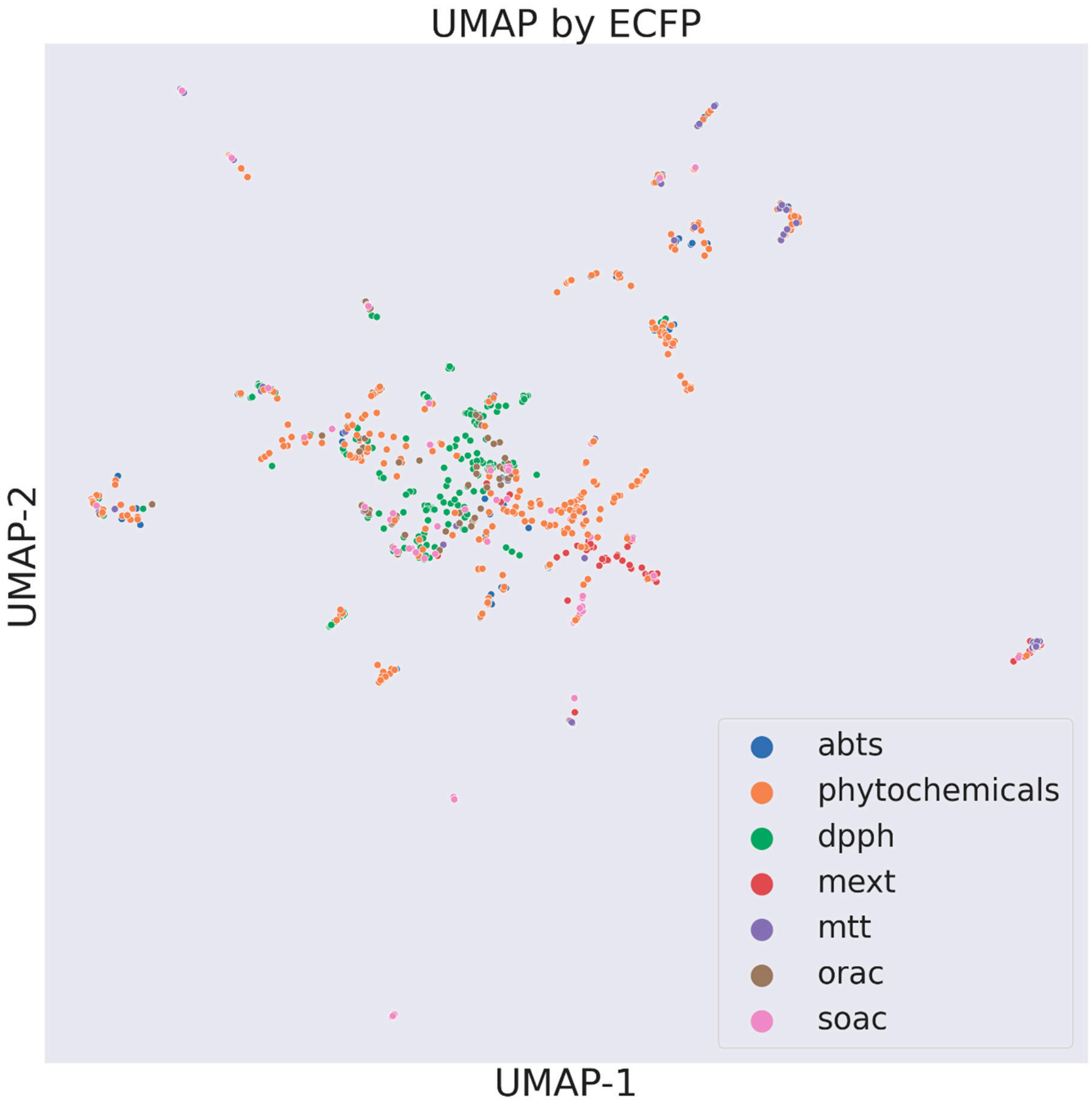

Figure S14 Distribution of antioxidants obtained from extended connectivity fingerprints by using UMAP. It is generated by ChemPlot [10].

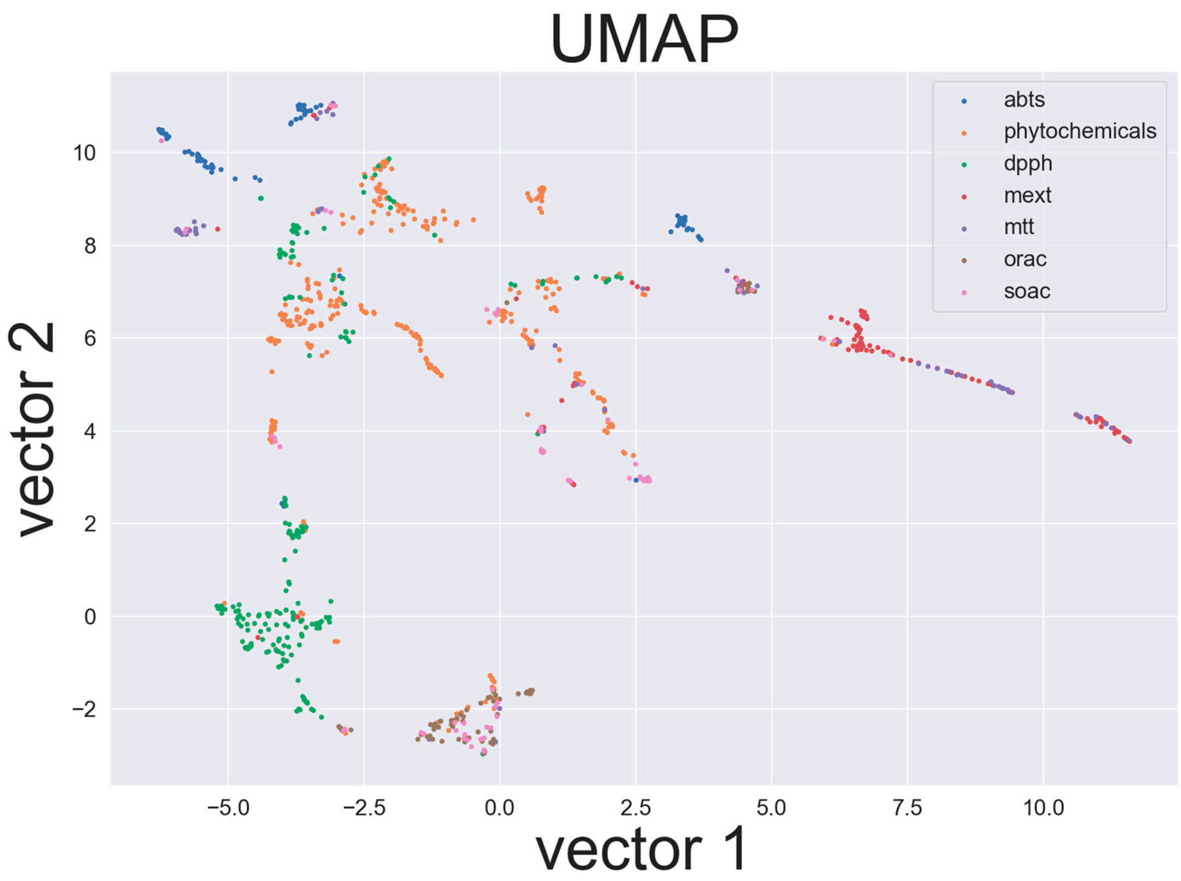

Figure S15 Distribution of antioxidants obtained from 15 features by using UMAP. Bond dissociation energy (BDE), ionization potential (IP), and proton affinity (PA) were excluded from 18 common features.

Reference

1. Sakurai, S.; Kikuchi, A.; Gotoh, H. Hydrophilic Oxygen Radical Absorbance Capacity Values of Low-Molecular-Weight Phenolic Compounds Containing Carbon, Hydrogen, and Oxygen. *RSC Adv* **2022**, *12*, 4094–4100, doi:10.1039/D1RA08918H.
2. Fujimoto, T.; Gotoh, H. Prediction and Chemical Interpretation of Singlet-Oxygen-Scavenging Activity of Small Molecule Compounds by Using Machine Learning. *Antioxidants* **2021**, *10*, 1751, doi:10.3390/antiox10111751.
3. Liu, Y.; Nair, M.G. An Efficient and Economical MTT Assay for Determining the Antioxidant Activity of Plant Natural Product Extracts and Pure Compounds. *J Nat Prod* **2010**, *73*, 1193–1195, doi:10.1021/np1000945.
4. Cai, Y.-Z.; Mei Sun; Jie Xing; Luo, Q.; Corke, H. Structure–Radical Scavenging Activity Relationships of Phenolic Compounds from Traditional Chinese Medicinal Plants. *Life Sci* **2006**, *78*, 2872–2888, doi:10.1016/j.lfs.2005.11.004.
5. Lu, A.; Yuan, S.; Xiao, H.; Yang, D.; Ai, Z.; Li, Q.-Y.; Zhao, Y.; Chen, Z.; Wu, X. QSAR Study of Phenolic Compounds and Their Anti-DPPH Radical Activity by Discriminant Analysis. *Sci Rep* **2022**, *12*, 7860, doi:10.1038/s41598-022-11925-y.
6. Phytochemicals | Tokyo Chemical Industry Co., Ltd.(JP) Available online: https://www.tcichemicals.com/JP/en/c/11115 (accessed on 3 November 2022).
7. MEXT : STANDARD TABLES OF FOOD COMPOSITION IN JAPAN - 2015 - (Seventh Revised Version) Available online: https://www.mext.go.jp/en/policy/science_technology/policy/title01/detail01/1374030.htm (accessed on 3 November 2022).
8. McInnes, L.; Healy, J.; Melville, J. UMAP: Uniform Manifold Approximation and Projection for Dimension Reduction. *J Open Source Softw* **2018**, 3, 861, doi:10.21105/joss.00861.
9. Rogers, D.; Hahn, M. Extended-Connectivity Fingerprints. *J Chem Inf Model* **2010**, *50*, 742–754, doi:10.1021/ci100050t.
10. Cihan Sorkun, M.; Mullaj, D.; Koelman, J.M.V.A.; Er, S. ChemPlot, a Python Library for Chemical Space Visualization. *Chemistry–Methods* **2022**, *2*, e202200005, doi:10.1002/cmtd.202200005.
